# Association of Wearable Device–Measured Step Volume and Variability With Blood Pressure in Older Chinese Adults: Mobile-Based Longitudinal Observational Study

**DOI:** 10.2196/50075

**Published:** 2024-08-14

**Authors:** Han Xiao, Zechen Zhou, Yujia Ma, Xiaoyi Li, Kexin Ding, Xiaotong Dai, Dafang Chen

**Affiliations:** 1 Department of Epidemiology and Biostatistics Peking University Beijing China; 2 Key Laboratory of Epidemiology of Major Diseases (Peking University) Ministry of Education Beijing China; 3 School of Sport Science Beijing Sport University Beijing China; 4 Key Laboratory of Ministry of Education for Sports and Physical Health Beijing Sport University Beijng China

**Keywords:** older adults, physical activity, step volume, step variability, blood pressure, wearable devices, mHealth apps, mobile health apps, mobile phone

## Abstract

**Background:**

The paucity of evidence on longitudinal and consecutive recordings of physical activity (PA) and blood pressure (BP) under real-life conditions and their relationships is a vital research gap that needs to be addressed.

**Objective:**

This study aims to (1) investigate the short-term relationship between device-measured step volume and BP; (2) explore the joint effects of step volume and variability on BP; and (3) examine whether the association patterns between PA and BP varied across sex, hypertension status, and chronic condition status.

**Methods:**

This study used PA data of a prospective cohort of 3070 community-dwelling older adults derived from a mobile health app. Daily step counts, as a proxy of step volume, were derived from wearable devices between 2018 and 2022 and categorized into tertiles (low, medium, and high). Step variability was assessed using the SD of daily step counts. Consecutive daily step count recordings within 0 to 6 days preceding each BP measurement were analyzed. Generalized estimation equation models were used to estimate the individual and joint associations of daily step volume and variability with BP. Stratified analyses by sex, the presence of hypertension, and the number of morbidities were further conducted.

**Results:**

A total of 3070 participants, with a median age of 72 (IQR 67-77) years and 71.37% (2191/3070) women, were included. Participants walked a median of 7580 (IQR 4972-10,653) steps and 5523 (IQR 3590-7820) meters per day for a total of 592,597 person-days of PA monitoring. Our results showed that higher levels of daily step volume were associated with lower BP (systolic BP, diastolic BP, mean arterial pressure, and pulse pressure). Compared with participants with low step volume (daily step counts <6000/d) and irregular steps, participants with high step volume (≥9500/d) and regular steps showed the strongest decrease in systolic BP (–1.69 mm Hg, 95% CI –2.2 to –1.18), while participants with medium step volume (6000/d to <9500/d) and regular steps were associated with the lowest diastolic BP (–1.067 mm Hg, 95% CI –1.379 to –0.755). Subgroup analyses indicated generally greater effects on women, individuals with normal BP, and those with only 1 chronic disease, but the effect pattern was varied and heterogeneous between participants with different characteristics.

**Conclusions:**

Increased step volume demonstrated a substantial protective effect on BP among older adults with chronic conditions. Furthermore, the beneficial association between step volume and BP was enhanced by regular steps, suggesting potential synergistic protective effects of both increased step volume and step regularity. Targeting both step volume and variability through PA interventions may yield greater benefits in BP control, particularly among participants with hypertension and a higher chronic disease burden.

## Introduction

### Background

Elevated blood pressure (BP) is a significant risk factor for cardiovascular disease and mortality and is highly prevalent in older adults [1,2]. The continuously increasing number of older individuals further leads to a growing population with high BP, which poses a significant need for both home and institutionalized BP management [1].

Evidence to date has recognized physical activity (PA) as a primary strategy for controlling BP [3,4]. For older adults, walking is the most common and feasible way of PA, which requires only a minimal level of fitness and can be done almost anywhere [5,6]. Previous epidemiological evidence has consistently shown that taking higher steps per day is associated with BP reduction and lower cardiovascular disease risk [2,3,7], implicating the potential of daily steps as a target for personalized exercise prescriptions in older adults. In accordance, the 2018 Physical Activity Guidelines Advisory Committee emphasized the importance of daily walking for its ability to improve the translation of research findings into public health recommendations [8].

However, previous studies highlighting the beneficial effects of walking are limited by their single assessments over short durations at baseline (eg, 7 days), which fail to capture consecutive daily step counts over time and may not be representative of habitual walking behavior in daily life. For example, most previous cohort studies, such as Toledo Study for Healthy Aging [9] and UK Biobank [10], measured participants’ daily step levels only within 1 week at baseline and then examined their associations with outcomes during long-term follow-up periods. Recognizing this limitation, a few recent observational studies have been conducted to monitor step counts over extended periods, typically spanning 6 months to 2 years, thereby accounting for longitudinal changes in step counts over time [7,11,12]. While these studies have yielded novel insights into the associations between step counts and cardiovascular risks, little is known about the temporal relationships between daily steps and subsequent BP. This gap in knowledge is partly attributable to the absence of consecutive daily assessments of cardiovascular outcomes in current study designs. Furthermore, despite regular PA being commonly recommended by guidelines [13], previous studies have yet to quantitatively assess the variability of PA or the joint effects of PA volume and variability on BP. Indeed, structured interventions implemented by randomized controlled trials often result in homogeneity in the regularity of PA, making it challenging to detect the effects of activity variability.

Monitoring individual PA and BP consecutively in real-world scenarios and over a long period is currently a knowledge gap. Digital health technologies, including consumer-based wearable devices and mobile health (mHealth) apps, present a valuable opportunity to bridge this gap through remote data recording, uploading, and management [14]. mHealth apps also have the potential to provide tailored health information, multidimensional health data aggregation, self-monitoring, and feedback service to the users, all of which would enhance participant adherence to longitudinal studies.

### Objectives

In this study with a focus on older adults, we analyzed a total of 592,597 person-days of PA records and 188,671 BP measurements collected from an mHealth app in China across a 5-year period. We aimed to (1) investigate the short-term relationship between device-measured step volume and BP; (2) explore the joint effects of step volume and variability on BP; and (3) examine whether the association patterns between PA and BP varied across sex, hypertension status, and chronic condition status. We hypothesized that older adults with different characteristics would exhibit varying responses to daily steps, and our findings have the potential to inform personalized remote health management strategies for aging populations.

## Methods

### Study Population

This analysis is based on a population-based cohort of community-dwelling older adults in China. Participants were recruited and managed via an mHealth app (YIDO Health app, YIDO Artificial Intelligence Technology Co, Ltd) in combination with consumer wearable devices. The YIDO mHealth app was commercially designed to provide personalized health information monitoring, health assessment, and health guidance, especially for older adults [15]. Upon system log in, users could connect wearable activity trackers to the smartphone and securely upload monitoring data to the web-based app. The analytic data were collected in an anonymous and aggregated data set without personal identifiers such as names, email addresses, or cell phone numbers. Random strings were used to identify the PA records for each day.

Retrospective analyses were conducted using 592,597 days of PA data and 188,671 BP recordings of 3070 participants from the mHealth app between 2018 and 2022. Eligible participants for the study were community-dwelling older adults who were users of the YIDO remote management system. Inclusion criteria included age ≥60 years, fluency in Chinese, ownership of a smartphone with a supported iOS or Android version, possession of disease history information, regular access to the app for uploading daily activity data throughout the study period, and informed consent before the study. Participants were excluded due to the following reasons: (1) their wrist or bicep circumference precluded comfortable use of the watch or BP cuff; (2) they had a clinical contraindication to noninvasive BP measurements; (3) incomplete step count or BP data; (4) step count or BP outliers; and (5) not having at least 7 consecutive days of step count recordings followed by at least 1 valid measurement of BP.

### Measurement

#### Step Volume and Variability Assessment

The participants were provided with a Lexin Mio smart bracelet (Mambo, Lexin). A Lexin Mio smart bracelet is a 3-axis acceleration sensor-based wearable device that tracks and monitors the user’s steps, walking distance, heart rate, and other biometric data. Its validity has been reported previously [16]. All participants were given specific instructions to wear their watches during the whole day (24 hours) and for as many days as possible. Evidence suggests that 10 hours of wear time is sufficient to estimate daily PA during waking time [17]. To ensure the completeness of tracking, we, therefore, defined a valid day as one when the device is worn for at least 16 hours and at least 250 steps are reported. The watch wear time accounted only for the minutes reporting a valid heart rate measurement.

The most frequently used and relevant metric to reflect one’s physical behaviors was the number of steps taken per day, hereon referred to as daily step count [18]. In this analysis, we computed (1) daily step counts and (2) SD of daily steps to assess PA characteristics. Daily step counts are considered as a reliable proxy of the overall volume of PA performed by a participant [14]. The SD of daily steps is considered here as an indicator of the variability of PA and as a proxy of regularity [19]. A low SD value indicates low variability and, therefore, a high regularity of the PA level. Daily step volume were categorized as low, medium, and high based on the tertile distributions of both the whole sample or specific subpopulations in terms of sex, hypertension status, or chronic morbidities status (Multimedia Appendix 1). The variability of daily steps was categorized based on the median value of the SD of daily steps (2000 steps/d): regular (SD of daily step counts<2000/d) and irregular (SD of daily step counts≥2000/d).

#### BP Monitoring

BP monitors (C02L, Heal Force) were provided to monitor BP during the study. To quantify BP, the user was instructed to (1) rest for at least 5 minutes before taking a measurement; (2) be seated in a comfortable position with legs uncrossed, feet flat on the floor, and back or arms supported; (3) not speak during the measurements; and (4) perform the measurement on their left arm. The resulting BP is uploaded to the user’s app account. Participants could record BP at any time of day convenient to them. A BP measurement was excluded if the systolic BP (SBP) was <50 mm Hg or >300 mm Hg or if the diastolic BP (DBP) was <30 mm Hg or >140 mm Hg. Then, mean arterial pressure (MAP) was calculated using the formula MAP = (SBP + [2 * DBP])/3. Pulse pressure (PP) was calculated as the difference between SBP and DBP.

### Covariates

Covariates were selected based on the literature and the biological plausibility for confounding the main associations of interest. Covariates obtained at baseline included sex, age, smoking status (never, ever, or current), and drinking status (never, ever, or current). BMI was calculated as weight (kg) divided by height squared (m^2^). The presence of hypertension and type 2 diabetes was ascertained by the self-report of physician diagnosis and the use of antihypertensive and antidiabetic medications. The number of morbidities, including asthma, arthritis, cancer, chronic obstructive pulmonary disease, chronic kidney disease, coronary heart disease, diabetes, hyperlipidemia, hypertension, osteoporosis, peptic ulcer, and stroke, was used to account for the comorbidity status of participants in the study [20]. The selection of conditions was based on the Centers for Disease Control and Prevention recommendations for defining and measuring multiple chronic conditions [21]. Tracking seasons were divided into quarters as spring (March to May), summer (June to August), autumn (September to November), and winter (December to February) in this study because of the large latitude span in China. In addition, the date of recording was used to define weekdays and weekends and to adjust for the long-term time trend.

### Statistical Analysis

#### Descriptive Analysis

Baseline characteristics of the study population were reported as median (IQR) for continuous variables and as numbers (percentages) for categorical variables.

#### Main Analyses on the Association of Step Volume and Variability With BP

**Figure 1:**
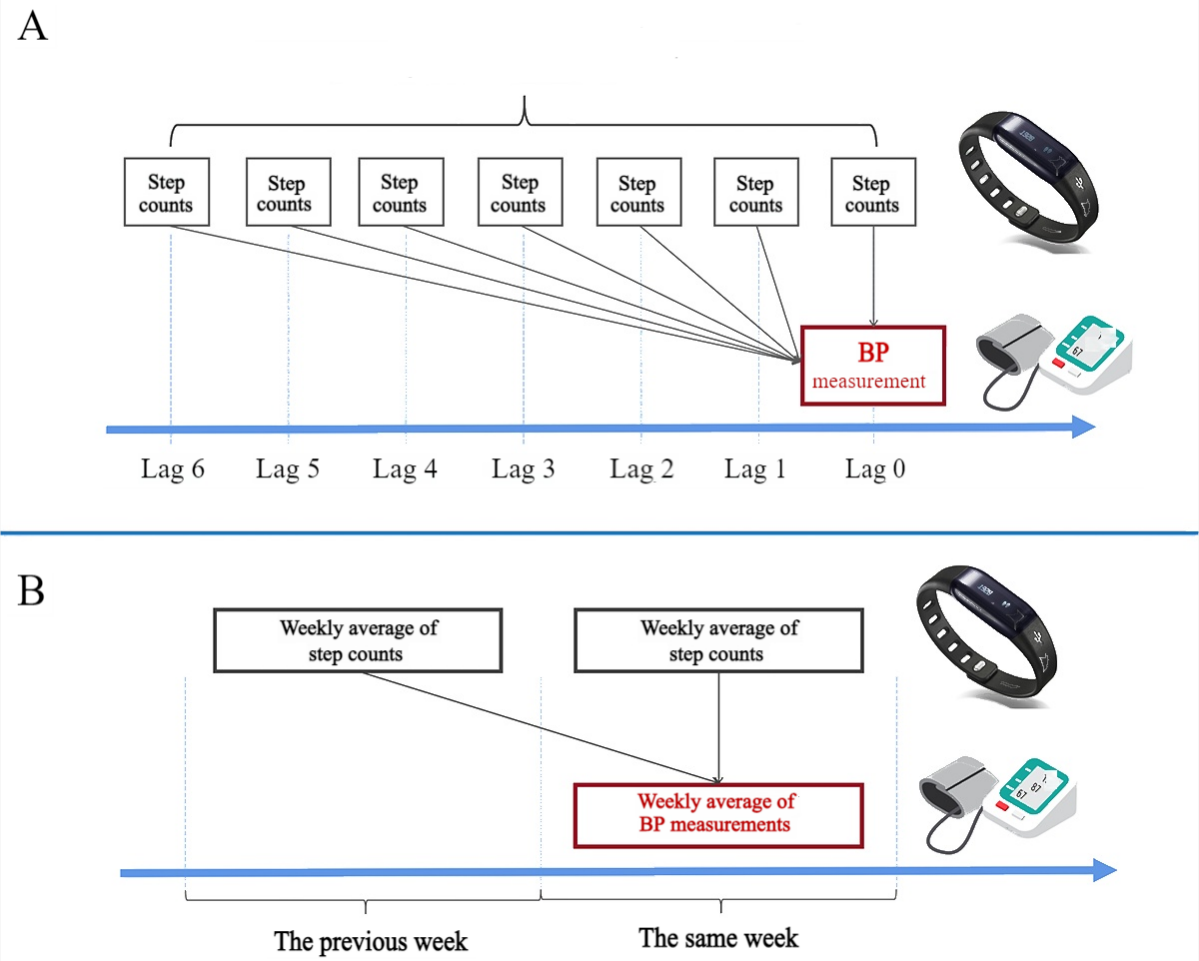
Study design for investigating the association of step volume and variability with blood pressure (BP). (A) Study design for main analyses. Physical activity (PA) recordings on the same day of BP measurement were considered as 0 days preceding the BP measurement and denoted as lag 0. In this manner, consecutive recordings of 0 to 6 days before every BP measurement were retrieved. The cumulative effects of PA were examined using the moving average value of step counts in lag 0 to 6 days. Step variability was computed as the SD of step counts in lag 0 to 6 days. (B) Study design for sensitivity analyses. Week-to-week associations were examined to reduce potential bias caused by large amounts of repeated measures and to support the credibility of our results. We focused on the weekly average PA 1 week before or on the same week of BP measurements. 1 valid week was defined as consisting of at least 3 days of BP measurement and at least 5 days of step count recordings.

Personal PA recordings were linked to the participants’ BP measurements according to the monitoring date. To fully explore the short-term lag structure, step count recordings on the same day as BP measurement were designated as lag 0, while those from the previous day were denoted as lag 1. This approach enabled the examination of the effects of walking steps with varying lag days from lag 0 to lag 6. Cumulative PA effects were further estimated using the 7-day moving average (lag 0 to lag 6) of step counts. Therefore, only participants with at least 7 consecutive valid daily walking records preceding each BP measurement were included in the analysis (Figure 1A).

Generalized estimating equation models were applied using daily step counts categorized into tertiles (low, medium, and high). The advantages of generalized estimating equation models are that they consider the within-individual correlation in exposure and outcome due to repeated measures. They can also better handle unbalanced data, as the number of repeated measures per participant can be different from one participant to another [18]. For all models, we adjusted for the following covariates: age, sex, BMI, smoking status (never, ever, or current), drinking status (never, ever, or current), history of hypertension, history of type 2 diabetes, season, and type of record day (weekday or weekend). We also adjusted the time trend by including a natural spline of calendar dates with 5 df. This model choice was governed by the quasi-likelihood under the independence model criterion values of models.

Participants were further categorized into 6 mutually exclusive groups to investigate the joint associations of weekly average step volume (low, medium, or high) and step variability (regular or irregular) with BP, with the group with low step volume and irregular steps as a reference. Proportions for each combined group are presented in Multimedia Appendix 2. The same models described earlier were used for data analysis.

We further conducted a series of stratified analyses by sex, the presence of hypertension, and the number of morbidities (with 1 morbidity, with 2 morbidities, and with at least 3 morbidities). When performing the stratified analyses, daily step volume and walking distance were categorized in tertiles based on the cut-offs of specific subpopulations (Multimedia Appendix 1). The joint associations of step volume and step variability across various subgroups were also examined using stratified analyses.

#### Sensitivity Analysis

A series of sensitivity analyses were performed. First, to prove the generalizability of our results, stratified analyses using categorized daily PA determined by tertiles of the whole population rather than based on subgroups were conducted. Second, the literature on using pedometers to measure PA suggests interpreting raw step counts rather than converting them to distance, although the basis for this recommendation is unclear [6]. As daily walking distance was also recorded by the wearable devices, we repeated the main analyses using walking distance as an indicator of daily PA. Third, given the large amount of repeated BP measures on the time scale of a day, we further designed week-to-week association analyses to reduce potential bias caused by repeated measures and support the credibility of our results. In this analysis, a valid week was defined as consisting of at least 3 days of BP measurements and at least 5 days of step counts and walking distance records. In addition, we considered the weekly average PA in the same week and in the previous week of BP measurement as exposure variables. Therefore, participants with 1 valid week of BP records within 2 consecutive valid weeks of PA records were considered for the analysis (Figure 1B). The analytic sample for this step was derived from the sample for the main analyses (Multimedia Appendix 3).

Statistical analyses were conducted using R (version 4.2.0; R Foundation for Statistical Computing). Significance levels were set at *P*<.05.

### Ethical Considerations

Participants provided electronic consent for participation upon creating their accounts and accepting the anonymous treatment of personal data by YIDO for research purposes. The extracted data were anonymized and managed according to privacy and data protection regulations. This study was conducted in compliance with the Declaration of Helsinki principles and approved by the Sports Science Experiment Ethics Committee of Beijing Sport University (number 2024012H). All patients gave written informed consent to participate in this study. They did not receive any financial or other compensation.

## Results

### Characteristics of the Study Population

The participants (N=3070) had a median age of 72 (IQR 67-77) years and a median BMI of 23.9 (IQR 22.2-25.8) kg/m^2^ (Table 1). Overall, 71.37% (2191/3070), 45.28% (1390/3070), and 57.26% (1758/3070) of the participants were female, had hypertension, and had at least 2 morbidities, respectively. This study comprised 188,671 accumulated person-days of BP measurements and 592,597 person-days of PA records over 1351 days of tracking. Participants monitored BP for a median of 34 (IQR 10-75) days. From the overall days studied, the median values of SBP, DBP, MAP, and PP were 125 (IQR 117-134) mm Hg, 71 (IQR 65-78) mm Hg, 89.3 (IQR 83-95) mm Hg, and 54 (IQR 46-62) mm Hg, respectively. Wearable devices for detecting PA were worn for a median of 161 (IQR 55-300) days, with participants taking a median of 7580 (IQR 4972-10,653) steps per day.

**Table 1:** Characteristics of the study population.

Characteristics			Overall (N=3070)	Female participants (n=2191)	Male participants (n=879)
Age (y), median (IQR)			72 (67-77)	71 (67-76)	74 (69-80)
BMI (kg/m^2^), median (IQR)			23.90 (22.23-25.78)	23.83 (22.19-25.78)	24.12 (22.31-25.76)
**Smoking status, n (%)**					
	Never		2622 (85.41)	2112 (96.39)	510 (58.02)
	Ever		303 (9.87)	66 (3.01)	237 (26.96)
	Current		145 (4.72)	13 (0.59)	132 (15.02)
**Drinking status, n (%)**					
	Never		2425 (79)	1995 (91.05)	430 (48.92)
	Ever		349 (11.37)	129 (5.89)	220 (25.03)
	Current		296 (9.64)	67 (3.06)	229 (26.05)
Hypertension, n (%)			1390 (45.28)	965 (44.04)	425 (48.35)
**Number of morbidities, n (%)**					
	1		1307 (42.57)	904 (41.26)	403 (45.85)
	2		803 (26.16)	567 (25.88)	241 (27.42)
	≥3		955 (31.11)	720 (32.86)	235 (26.73)
**BP^a^ measurements**					
	Systolic BP (mm Hg), median (IQR)		125 (117-134)	125.00 (116.50-133.00)	126.00 (118.00-135.00)
	Diastolic BP (mm Hg), median (IQR)		71 (65-78)	70.50 (64.00-77.00)	72.00 (66.00-79.00)
	Mean arterial pressure (mm Hg), median (IQR)		89.33 (83-95)	89.00 (82.67-94.67)	90.33 (84.33-96.00)
	Pulse pressure (mm Hg), median (IQR)		54 (46-62)	54.00 (46.00-62.00)	53.00 (45.00-61.00)
	Accumulative BP measurements (days), n		188,671	132,464	56,207
	Measurements per person (days), median (IQR)		34 (10-75)	33 (10-74)	35 (12-76)
**Physical activity records**					
	Daily step counts (steps), median (IQR)		7580 (4972-10,653)	7545 (4975-10,546)	7672 (4964-10,923)
	**Daily step volume groups, n (%)**				
		Low	956 (31.14)	660 (30.12)	296 (33.67)
		Medium	1297 (42.25)	952 (43.45)	345 (39.25)
		High	817 (26.61)	579 (26.43)	238 (27.08)
	SD of daily step counts (steps), median (IQR)		2095 (1475-2854)	2109 (1499-2858)	2061 (1418-2843)
	Daily walking distance (meters), median (IQR)		5523.76 (3590-7820)	5480 (3573.85-7714.56)	5640 (3630-8100)
	**Daily walking distance groups, n (%)**				
		Low	1002 (32.64)	703 (32.09)	299 (34.02)
		Medium	1278 (41.63)	936 (42.72)	342 (38.91)
		High	790 (25.73)	552 (25.19)	238 (27.08)
	SD of daily walking distance (meters), median (IQR)		1564 (1093-2148)	1571 (1110-2149)	1545 (1058-2147)
	Total days of tracking (days), n		1351	1279	1301
	Accumulative activity records (days), n		592,597	417,886	174,711
	Records per person (days), median (IQR)		161 (55-300)	156 (51-293)	167 (63-309)
	**Type of the daily records tracked in, n (%)**				
		Weekdays	423,119 (71.4)	298,280 (71.4)	124,839 (71.5)
		Weekends	169,478 (28.6)	119,606 (28.6)	49,872 (28.5)
	**Seasons of records tracked in, n (%)**				
		Spring (March to May)	174,169 (29.4)	122,208 (29.2)	51,961 (29.7)
		Summer (June to August)	196,272 (33.1)	139,230 (33.3)	57,042 (32.6)
		Autumn (September to November)	98,215 (16.6)	68,834 (16.5)	29,381 (16.8)
		Winter (December to February)	123,941 (20.9)	87,614 (21.0)	36,327 (20.8)

^a^BP: blood pressure.

### Association of Step Volume With BP

**Figure 2:**
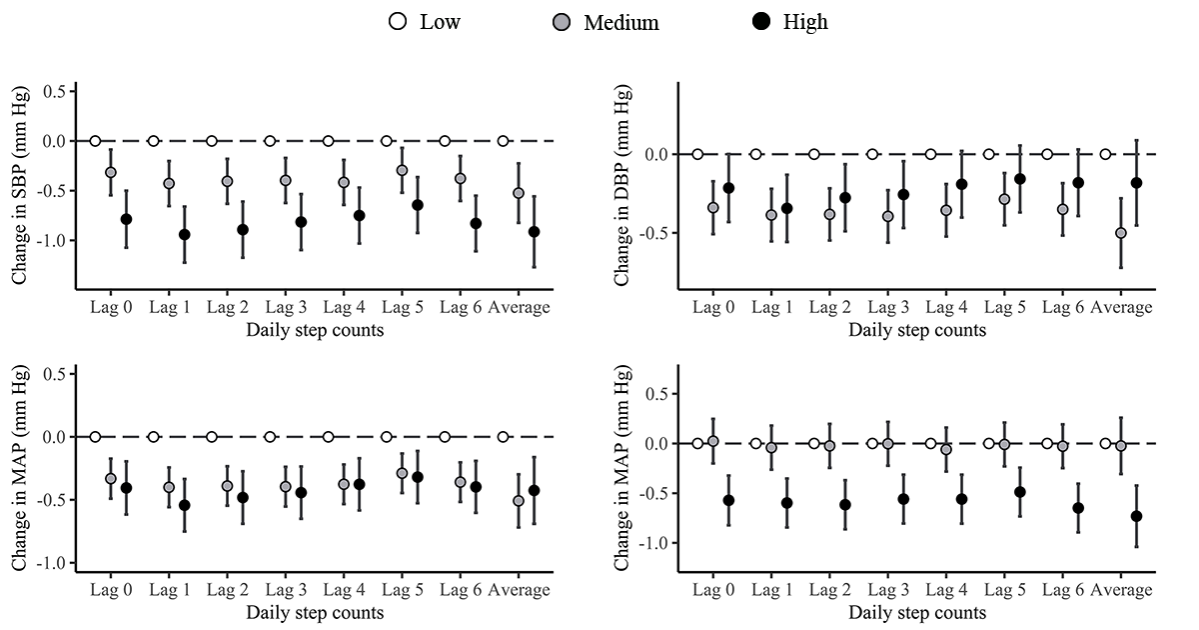
Estimated changes (95% CIs) in blood pressure (BP; systolic BP [SBP], diastolic BP [DBP], mean arterial pressure [MAP], and pulse pressure [PP]) related to daily step volume in different lag days and week averages. Step volume levels were categorized based on the tertiles of the whole sample: low (daily step counts <6000/d), medium (6000/day to <9500/d), and high (≥9500/d). Models are adjusted for age, sex, BMI, smoking status, drinking status, the presence of hypertension, the presence of type 2 diabetes, season, the type of day (weekday or weekend), and long-term trend.

Compared with participants with low daily step volume (<6000 steps/d), those with higher daily step volume exhibited lower BP levels. Notably, distinctive association patterns emerged across various BP metrics. For SBP, higher daily step volume were associated with significantly lower BP values. Conversely, for DBP, the most pronounced association with step volume was observed within the medium tier of step volume, while the association attenuated for the highest step volume level (Figure 2; Multimedia Appendix 4).

For the lag structure, the step volume on the previous day (lag 1) of BP measurement showed the greatest association with BP. This association substantially attenuated at lag s of 2 to 5 days before modestly increasing again for PA effects a week prior (lag 6). We further examined the cumulative effects of PA on BP by using the moving average step volume of lag 0 to 6 days, and we found that the cumulative effects were stronger than the effect produced by a single-day exposure. Compared with a low weekly average step volume, the medium and high levels of weekly average step volume had 0.524 (95% CI –0.822 to –0.226) mm Hg and 0.913 (95% CI –1.27 to –0.556) mm Hg decreases in SBP and 0.5 (95% CI –0.721 to –0.279) mm Hg and 0.182 (95% CI –0.452 to –0.088) mm Hg decreases in DBP, respectively (Figure 2; Multimedia Appendix 4).

### Joint Association of Step Volume and Variability With BP

#### Overview

**Figure 3:**
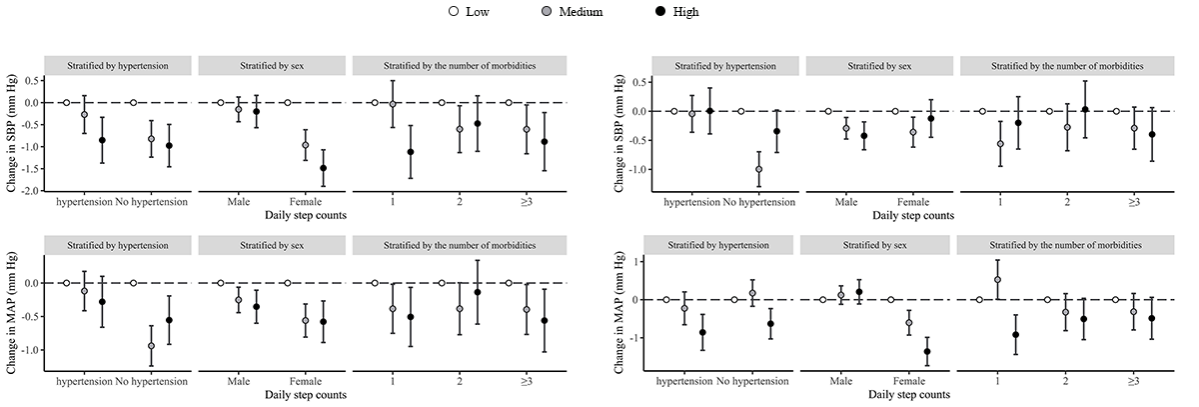
Estimated changes (95% CIs) in blood pressure (BP; systolic BP [SBP], diastolic BP [DBP], mean arterial pressure [MAP], and pulse pressure [PP]) related to joint effects of daily step volume and variability. Step volume levels were categorized based on the tertiles of the whole sample: low (daily step counts <6000/d), medium (6000/d to <9500/d), and high (≥9500/d). Step variability was categorized into regular (SD of daily step counts<2000/d) and irregular (≥2000/d). Models were adjusted for age, sex, BMI, smoking status, drinking status, the presence of hypertension, the presence of type 2 diabetes, season, the type of day (weekday or weekend), and long-term trend.

The joint association estimates for each condition of step volume and variability combinations compared with the referent group with low step volume and irregular steps are illustrated in Figure 3 and Multimedia Appendix 5. Generally, participants with a more regular step pattern had a stronger effect on BP than participants with an irregular step pattern, irrespective of their step volume level. Furthermore, among participants with regular steps in a week, those who had this in combination with a high level of step volume showed the strongest association with SBP, MAP, and PP, whereas the effects on DBP were the most pronounced for medium level of daily step volume. Specifically, participants with high step volume and regular steps showed the strongest decrease in SBP (–1.69 mm Hg, 95% CI –2.2 to –1.18), MAP (–1.019 mm Hg, 95% CI –1.407 to –0.631), and PP (–1.006 mm Hg, 95% CI –1.455 to –0.557), while participants with medium step volume and regular steps were associated with the lowest DBP (–1.067 mm Hg, 95% CI –1.379 to –0.755).

#### Subgroup Analyses of the Association of Step Volume and Variability With BP

**Figure 4:**
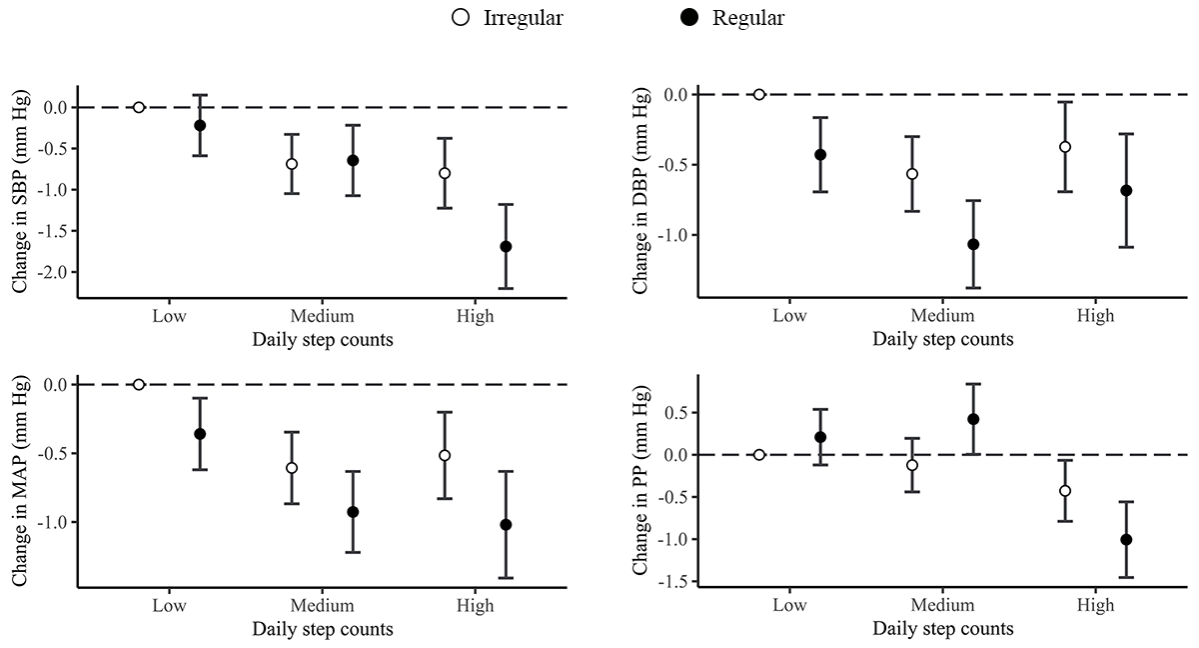
Estimated changes (95% CIs) in blood pressure (BP; systolic BP [SBP], diastolic BP [DBP], mean arterial pressure [MAP], and pulse pressure [PP]) related to the weekly average daily step volume level across sexes, the presence of hypertension, and chronic disease status. Daily step volume were categorized as low, medium, and high based on the tertile distributions of specific subpopulations. Models were adjusted for age, sex, BMI, smoking status, drinking status, the presence of hypertension, the presence of type 2 diabetes, season, the type of day (weekday or weekend), and long-term trend.

**Figure 5:**
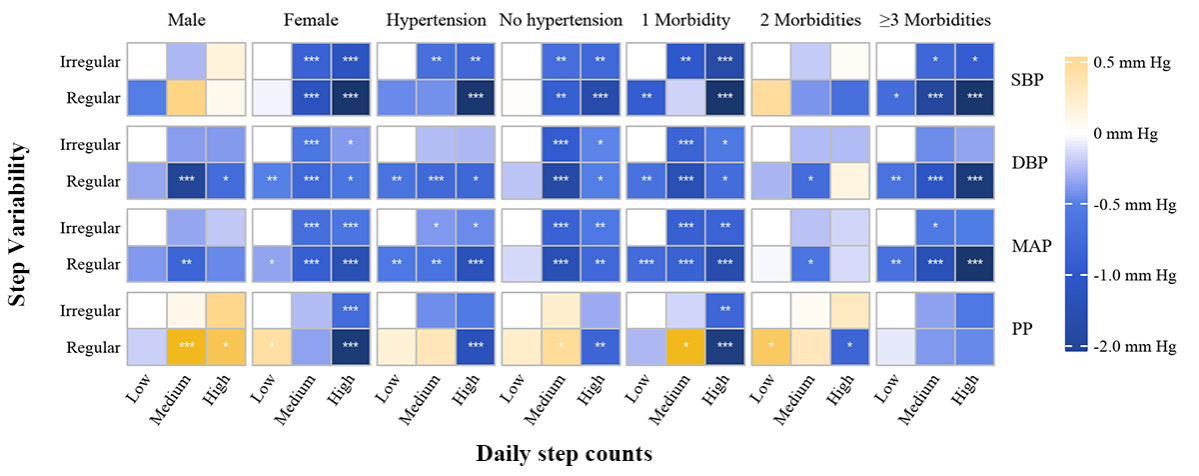
Joint associational patterns for step volume and variability with blood pressure (BP; systolic BP [SBP], diastolic BP [DBP], mean arterial pressure [MAP], and pulse pressure [PP]) across sexes, the presence of hypertension, and chronic disease status. Heatmap illustrated magnitudes in distinct patterns for the effects of physical activity (volume and variability) on BP across different subgroups. Daily step volume were categorized as low, medium, and high based on the tertiles of specific subpopulations. The SD of step volume over a week was categorized into regular (SD of daily step counts<2000/d) and irregular (≥2000/d). Models were adjusted for age, sex, BMI, smoking status, drinking status, the presence of hypertension, the presence of type 2 diabetes, season, the type of day (weekday or weekend), and long-term trend. *P<.05, **P<.01, ***P<001.

In stratified analyses, step volume and variability still had substantial associations with BP in different subgroups, which were generally consistent with the results among the overall population. Despite the general beneficial relationship between PA and BP, modification effects between subgroups were also observed (Figures 4 and 5; Multimedia Appendices 6 and 7). When classified by sex, the associations of step volume and variability with BP were more apparent in female participants. Higher step volume was associated with lower DBP and MAP for both sexes, whereas it was linked to lower SBP and PP for only women.

In terms of the presence of hypertension, the effects of step volume were more pronounced in participants without hypertension. Among patients with hypertension, only those in the high tertile of step volume demonstrated significantly lower SBP and PP (for SBP: –0.852 mm Hg, 95% CI –1.371 to –0.333; for PP: –0.871 mm Hg, 95% CI –1.345 to –0.397).

Considering the number of chronic morbidities, we observed that the effects of PA on BP were more remarkable in participants with only 1 chronic disease. For example, a high step volume decreased SBP by 1.1 mm Hg (95% CI –1.696 to –0.504) in participants with 1 morbidity, while the decrease was by –0.885 mm Hg (95% CI –1.546 to –0.224) for participants with at least 3 morbidities.

Finally, joint effects of the combination of step volume and variability were further investigated among subgroups (Figure 5). Similar to the overall population, we found that the beneficial associations between step volume and BP were exacerbated by regular steps, suggesting likely synergistic protective effects of step volume and variability. Interestingly, although step volume solely seemed not to be associated with DBP and MAP among patients with hypertension, participants combination of regular steps and high step volume had significantly lower DBP (–0.789 mm Hg, 95% CI –1.417 to –0.179) and MAP (–1.190 mm Hg, 95% CI –1.782 to –0.598) compared to participants with irregular steps and low step volume. These results indicated that step volume and variability may jointly affect BP. In addition, the associations between steps and BP would vary among older adults with different demographic characteristics or disease statuses.

#### Sensitivity Analyses

We conducted several sensitivity analyses, as described earlier, to ensure the credibility of our results. First, we used daily walking distance as another indicator of PA and repeated the main analyses (Figures S1 and S2 in Multimedia Appendix 8; Multimedia Appendices 4-7). The results were generally consistent with the results using daily step volume. It is worth noting that, for some subpopulations, the significance increased when using walking distance as an independent variable (Figure S3 in Multimedia Appendix 8). For instance, for participants with at least 3 morbidities, higher daily walking distance was significantly associated with lower DBP, which was not pronounced in terms of daily step volume. Second, to prove the generalizability of our results, we repeated the stratified analyses by categorizing step volume according to the tertiles of the whole population rather than specific subpopulations. The results were generally similar to the overall results (Multimedia Appendices 9 and 10). Finally, we conducted a week-to-week association analysis to reduce repeated measures of outcomes and exposures by calculating weekly averages of BP and step volume (Multimedia Appendices 11 and 12). The week-to-week association results were still consistent with the week-to-day association results, even though the association magnitudes were relatively lower. In addition, we found that the PA of the previous week showed greater effects on BP, compared with the PA of the same week.

## Discussion

### Principal Findings

To the best of our knowledge, this is one of the few studies to investigate the short-term benefits of PA. Using wearable device–measured daily step data from the YIDO mHealth app across 5 years and over 0.5 million person-days, the principal findings of our study are 3-fold. First, among community-dwelling older adults, we observed consistent negative associations between daily step volume and BP (SBP, DBP, MAP, and PP). Second, we noted a potential synergistic effect of step volume and variability on BP. Specifically, compared with irregular steps, regular steps appeared to improve the beneficial relationships between step volume and BP. Third, stratified analyses suggested that the effects of step volume and variability on BP varied across different subpopulations. When considering both step volume and variability jointly, we observed diverse associational patterns between PA and BP among older adults. Overall, our findings provided a comprehensive evaluation of the relationships of step volume and variability with BP among older adults. We underscored that a “one-size-fits-all” recommendation for step volume may not be suitable for all older adults.

### Independent Association Between Step Volume and BP

Previous studies have reported inconsistent evidence on the association between step volume and BP levels [4,22-25]. In a cross-sectional sample from the Electronic Framingham Heart Study, reductions in SBP of 0.49 mm Hg and DBP of 0.36 mm Hg were found per 1000 steps of daily step volume [25]. In comparison, a study of community-dwelling older participants did not observe a significant association between step counts and either SBP or DBP [4]. We postulated that prior studies with controversial results are limited by the short and cross-sectional monitoring periods of PA, which are prone to an observer effect and may not accurately reflect long-term PA behavior in real-world scenarios [26]. The development in digital health technologies made it feasible to remotely detect PA and BP continuously and longitudinally in a free-living environment. By using mHealth technologies, this is the first study to track the PA status of older adults in their daily lives for 5 years. Our findings contributed to the existing evidence that higher step volume is associated with lower BP (SBP, DBP, MAP, and PP).

A unique aspect of our findings was that the associational patterns varied between BP metrics. Specifically, our findings suggested that a high level of step volume showed the strongest beneficial effects on SBP and PP (≥9500 steps/d). In comparison, for DBP and MAP, the most significant association appeared in participants with a medium level of step volume (6000-9500 steps/d). Similarly, previous studies have reported that the relationship between step counts and adverse events may reverse as the step counts increase [5,10]. For example, the UK Biobank study, which quantified steps over a 7-day monitoring period and assessed mortality over a median of 7.0 years, found that a higher daily step count was associated with a lower mortality risk, whereas the risk increased after taking >10,000 steps per day [10]. We hypothesized that the divergent associational patterns between various BP metrics might be attributable to their physiological disparities: higher SBP and PP levels primarily reflect arterial stiffness in older adults, whereas DBP reflects coronary flow reserve [27]. Considering the varying patterns between BP metrics, we suggest the individualization of step targets depending on which BP metrics older adults were most burdened with.

The lag structure for the short-term effects was also investigated in this study. We observed that the step volume on the previous day of BP measurement was the most significantly associated with BP levels. In addition, it showed that cumulative effects, represented by the weekly average of daily step volume, had greater effects on BP than single-day exposure. A recent study showed that increasing PA levels were linked to blunted glycemic excursions during the next day, which was similar to our results [18]. A clinical trial study with 21 adults with prehypertension showed that SBP and DBP were significantly reduced after PA for 11 hours and 10 hours, respectively [28]. Another clinical trial observed that PA has antihypertensive effects within approximately 45 minutes, and these effects increase with the intensity of the exercise [29]. Possible mechanisms underlying the BP-lowering short-term effects of PA involve a reduction in sympathetic activity, lower insulin resistance due to the release of vasodilatory substances, and a decrease in plasma renin levels [30]. From a clinical perspective, short-term (24-hour) elevations in ambulatory BP have been identified as increasing the acute risk of cardiovascular disease events, including stroke and myocardial infarction [31]. Therefore, it is plausible that interventions aimed at improving daily step counts could have meaningful clinical implications in promoting the overall cardiovascular health, particularly among older adults.

### Joint Association of Step Volume and Variability With BP Level

Our study also provides novel estimations of the joint associations of step volume and variability with BP. In our study, participants taking more and regular steps per day had lower levels of BP, compared to those taking fewer and irregular steps. Although current PA guidelines generally recommend regular PA for improving fitness [8,13], few studies have investigated the effects of PA variability quantitatively or the synergistic effects of PA volume and variability quantitatively. There is only 1 recent study that reported that the greater regularity of the PA level may play an important role in short-term weight loss [14]. We believe the limited evidence on PA variability is because most previous randomized controlled trials have been based on structured interventions, where regularity is naturally part of the intervention. It is also difficult for observational studies to track the regularity of PA in daily lives based on a short period of PA monitoring.

In this study, we found that individuals with high step volume and regular steps experienced a notable decrease in SBP and DBP by 1.69 mm Hg and 0.69 mm Hg, respectively, compared to those with low step volume and irregular steps. These findings hold clinical significance, as they align with the results of a comprehensive meta-analysis evaluating the efficacy of antihypertensive agents in cardiovascular disease prevention. The meta-analysis demonstrated that reductions in SBP by 1 mm Hg and DBP by 0.5 mm Hg were associated with a 4% decrease in the incidence of stroke and a 2% decrease in coronary heart disease events [32]. Additional meta-analyses also showed that, at the population level, even an SBP decrease of 1 mm Hg could lower the risk of stroke by 5% [33]. Given the comparable magnitude between our observed BP reductions and the beneficial effects demonstrated in the previous meta-analyses, although the short-term association coefficients in our study may appear modest, they hold relevance from a public health perspective.

The concept of variability in other health-related indicators or behaviors has been extensively studied. For example, studies have investigated variability in glycemia [34]; BP [35]; and, more recently, sleep patterns [36,37]. Such research reveals that variability in health-related domains can have detrimental effects on health outcomes, where biological rhythm disruptions are often implicated in explaining these associations [36]. Accordingly, we supposed that the observed associations of step variability with BP may be explained by a similar mechanism: irregular step patterns serve as a marker of irregular health behaviors in general, including irregular timing of meals or exercise, which may contribute to circadian disruption [38,39]. Therefore, it is plausible that regular step patterns could contribute to the maintenance of balanced rest-activity rhythms and homeostatic physiological systems, thereby promoting the beneficial effects of step volume. Experimental studies are warranted to examine the potential causal mechanisms underpinning the detected relationships of PA volume and variability with BP.

### Stratified Analyses of the Association of Step Volume and Variability With BP

When classified by sex, the association of step volume and variability with BP seemed to be more apparent among female participants. For male participants, higher step volume was associated with lower DBP and MAP, although this association was not as pronounced for SBP and PP. Our findings are consistent with previous literature investigating the sex-related differences in the association of PA with BP. Previous epidemiological studies reported greater PA-associated reductions in BP among women than among men [40,41]. A systematic review provided further confirmation that compared to male individuals, female individuals experience a slightly greater reduction in both SBP and DBP due to the effects of walking [42]. The sex differences in the cardiovascular system persisted throughout life. Given that the sympathetic nervous system is more active in postmenopausal women [43,44], a possible explanation is that women may be more susceptible to the effects of PA on inducing neural network plasticity, which reduces sympathetic excitation [45].

Hypertensive effects were also investigated in our study. More significant associations between step volume and BP were observed in the population without hypertension. This observation is consistent with previous research indicating stronger effects of PA on BP in individuals with normal BP [41]. One possible explanation for this discrepancy may involve vessel wall thickening and remodeling in individuals with established hypertension, as arterial stiffness is often accompanied by a reduced sensitivity to vasodilatory effects. Therefore, the vasodilatory efficacy of PA may be greater in individuals with normal BP with elastic arteries than in individuals with hypertension with stiff arteries and advanced atherosclerotic wall changes [46]. Notably, although significant associations between step volume alone and DBP or MAP were not observed among patients with hypertension, pronounced effects were evident when considering the combination of step volume and variability. This suggests that individuals with hypertension may be particularly sensitive to the regularity of PA, emphasizing the importance of considering both the volume and regularity of exercise when establishing step targets for older individuals with hypertension.

Another innovation of our study lies in stratification according to the number of morbidities. Older adults commonly experience multiple chronic conditions that cause great burdens and costs [47]. Consistent with this, our study found that all participants had at least 1 chronic disease. However, present guidelines on PA offer uniform recommendations for all adults aged >65 years, irrespective of their chronic conditions [8,13]. Our findings suggested that step volume was inversely associated with BP across all subgroups with varying numbers of morbidities, with greater impacts in participants with only 1 morbidity. Participants with a great chronic disease burden may experience other pathogenetic pathways that more significantly affect the fluctuation of BP, such as oxidative stress and mitochondrial dysfunction [48], thus obscuring the impact of steps on BP. When evaluating the joint effects of volume and variability, the associations became more apparent in patients with >2 morbidities, possibly due to their increased sensitivity to PA regularity, as discussed earlier. Overall, our results underscore heterogeneous associational patterns among older adults with diverse chronic conditions, warranting personalized activity prescriptions for the older population.

### Strengths and Limitations

The main strengths of our study include a relatively large sample of community-dwelling older adults and longitudinal and continuous recordings of their PA in free-living conditions over 5 years. Wearable devices were used to measure daily steps and walking distance, which helped avoid participant errors in self-reported PA and accounted for the steps involved in any type of ambulatory activity [9]. To our knowledge, this is the first study estimating the association between step volume and BP over the entirety of an individual’s monitoring period rather than a brief snapshot. We also innovatively highlighted the joint effects of step volume and step variability on BP. Moreover, multifaceted subgroup analyses were conducted to compare discrepancies in the effects of PA on BP according to different population characteristics.

Some limitations to our study must be considered in interpreting the findings. First, the observational nature of our data precludes a definitive causal interpretation of our findings, although we took several measures to minimize risks for reverse causalities and confounding, such as including a wide range of covariates and conducting sensitivity analyses. The precise mechanisms explaining the short-term relationships between PA and BP should be assessed using different study designs. Second, there were significant differences in some characteristics between retained individuals and those excluded because of missing data. In addition, the study sample comprised only individuals who uploaded their PA and BP data as users of the YIDO app, for which further selection biases may exist. Thus, the generalizability of our results to nonretained individuals may be limited, although the average step counts of our participants were similar to those reported in prior studies involving Chinese older adults [2,49]. Third, there is a potential for unmeasured confounding in our analyses. For example, we were not able to track the BMI status of participants over time. Furthermore, information on individual dietary (eg, alcohol consumption) was not available, so we could neither control for them in our analyses nor assess their potential relative contribution to PA and BP change. Further investigations with complementary data on individual diets are warranted to address these gaps. Fourth, the 3-axis acceleration used by the commercial wearable device to measure step count has been reported to have high degrees of accuracy for step counts [50]. However, evidence demonstrating the validity and reliability of step counts measured using the Lexin Mio smart bracelet is limited, so additional studies are needed to investigate possible measurement errors. Step counts can vary widely by device brand and algorithms or analytical decisions [2]. Finally, PA intensity or cadence (steps/min) was not accessible in this study, as we had access only to daily step data. Nonetheless, daily step count is an increasingly widespread and intuitive metric that can be easily used as a target for health benefits.

### Conclusions

This study extends the contemporary evidence confirming that a greater number of daily steps is significantly associated with a lower level of BP (SBP, DBP, MAP, and PP). In addition, we observed that step volume and variability are jointly associated with BP. Participants with high step volume and regular steps had lower BP compared with participants with low step volume and irregular steps. Subgroup analyses further indicated heterogeneous associational patterns among older adults with various chronic conditions. For participants with hypertension and with more chronic disease burden, PA interventions combining both step volume and variability can result in more benefits in controlling BP. Collectively, these results provide an evidence base for refining activity recommendations based on an individual’s characteristics. This study also provides an example of the potential clinical value of the integration of consumer wearable devices and mHealth apps.

## Appendix

Multimedia Appendix 1 Cut-off values of daily step volume across different groups.

Multimedia Appendix 2 Numbers and proportions for the combined groups.

Multimedia Appendix 3 Characteristics of the study population for week-to-week associations.

Multimedia Appendix 4 The independent associations of daily step volume and daily walking distance with blood pressure (systolic blood pressure, diastolic blood pressure, mean arterial pressure, and pulse pressure).

Multimedia Appendix 5 The joint associations of physical activity (daily steps and walking distance) volume and variability with blood pressure (systolic blood pressure, diastolic blood pressure, mean arterial pressure, and pulse pressure).

Multimedia Appendix 6 Stratified analyses for the independent associations of daily step volume and daily walking distance with blood pressure (systolic blood pressure, diastolic blood pressure, mean arterial pressure, and pulse pressure).

Multimedia Appendix 7 Stratified analyses for the joint associations of physical activity (daily steps and walking distance) volume and variability with blood pressure (systolic blood pressure, diastolic blood pressure, mean arterial pressure, and pulse pressure).

Multimedia Appendix 8 Estimated changes (95% CIs) in blood pressure (BP; systolic BP, diastolic BP, mean arterial pressure, and pulse pressure) related to daily walking distance in different lag days and week averages, joint effects of daily walking distance and variability, and weekly average daily step volume and walking distance across gender, the presence of hypertension, and chronic disease status.

Multimedia Appendix 9 Stratified analyses for the independent associations of daily step volume and daily walking distance with blood pressure (systolic blood pressure, diastolic blood pressure, mean arterial pressure, and pulse pressure), categorized according to the tertiles of the whole population.

Multimedia Appendix 10 Stratified analyses for the joint associations of physical activity (daily steps and walking distance) volume and variability with blood pressure (systolic blood pressure, diastolic blood pressure, mean arterial pressure, and pulse pressure), categorized according to the tertiles of the whole population.

Multimedia Appendix 11 Independent associations of daily step volume and daily walking distance with blood pressure (systolic blood pressure, diastolic blood pressure, mean arterial pressure, and pulse pressure) in week-to-week association patterns.

Multimedia Appendix 12 Joint associations of physical activity (daily steps and walking distance) volume and variability with blood pressure (systolic blood pressure, diastolic blood pressure, mean arterial pressure, and pulse pressure) in week-to-week association patterns.

## Abbreviations

BP: blood pressure
DBP: diastolic blood pressure
MAP: mean arterial pressure
mHealth: mobile health
PA: physical activity
PP: pulse pressure
SBP: systolic blood pressure

## Data Availability

The data sets used or analyzed during this study are available from the corresponding author upon reasonable request.
