# Supplementary material for: Association of Wearable Device–Measured Step Volume and Variability With Blood Pressure in Older Chinese Adults: Mobile-Based Longitudinal Observational Study
Source: J Med Internet Res. 2024 Aug 14;26:e50075. doi: 10.2196/50075 (PMC11358660; doi:10.2196/50075)
Supplement: Multimedia Appendix 8 [file jmir_v26i1e50075_app8.docx]

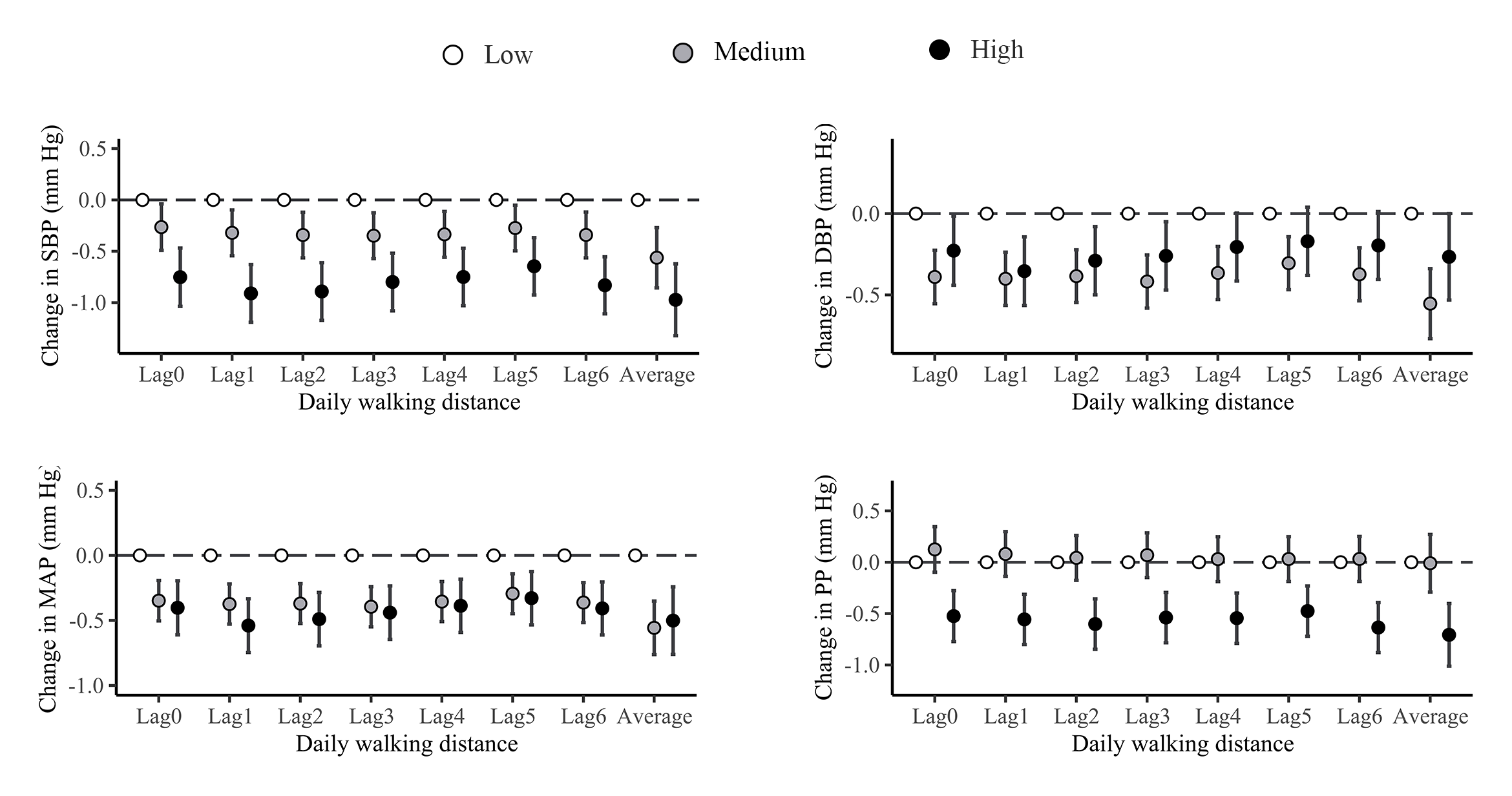


Figure S1. Estimated changes (95% confidence intervals) in blood pressure (SBP, DBP, MAP, PP) related to daily walking distance level in different lag days and week average. Daily walking distance was categorised based on the terciles of the whole sample: low (daily step counts $<$4.5 km/day); medium (4.5 km/day to $<$7 km/day); high ($\geq$7 km/day). Models are adjusted for age, sex, body mass index, smoking status, drinking status, presence of hypertension, presence of type 2 diabetes, season, type of the day (weekday/weekend), and long term trend. SBP, systolic blood pressure; DBP, diastolic blood pressure; MAP, mean arterial pressure; PP, pulse pressure.


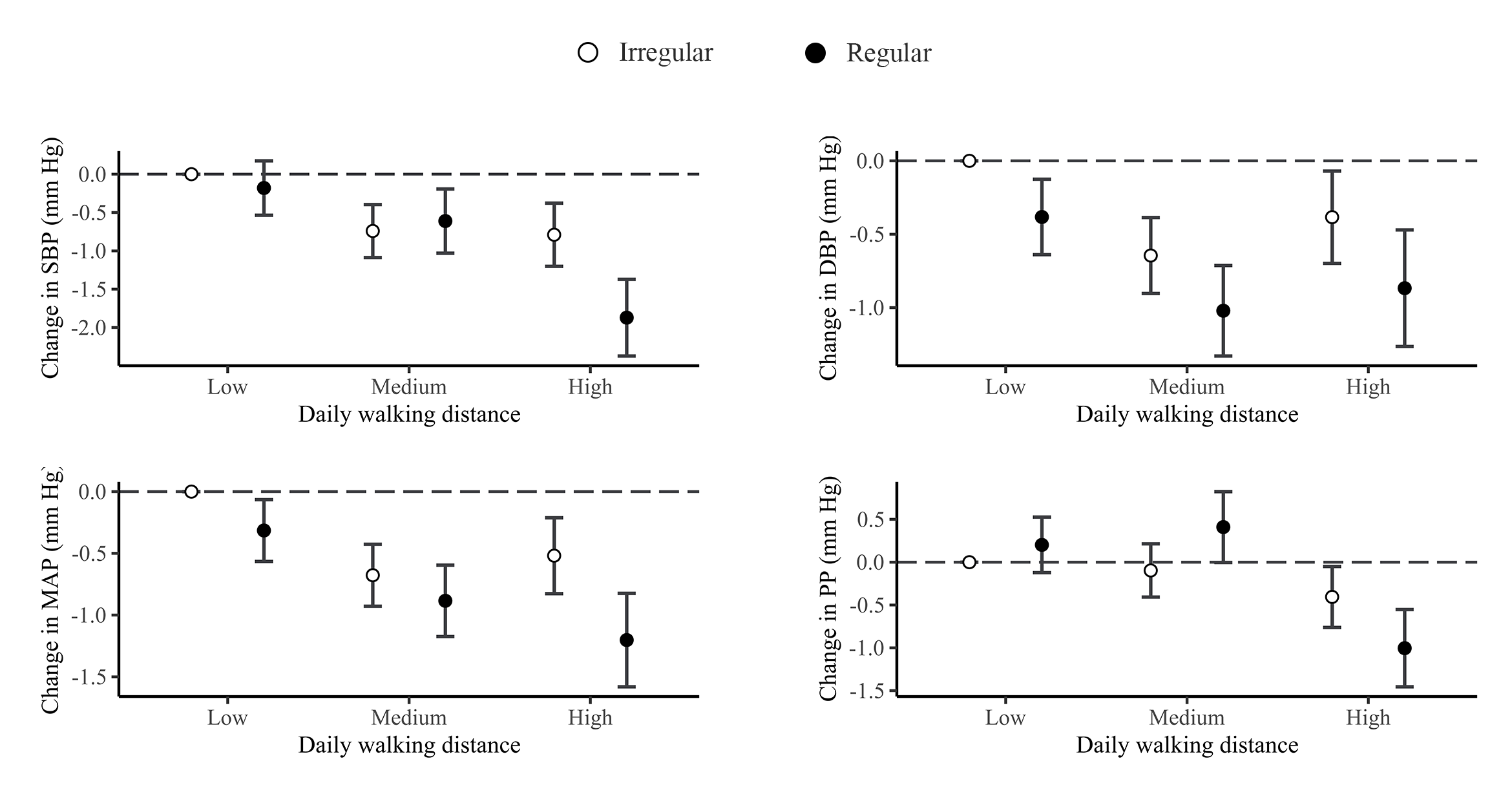


Figure S2. Estimated changes (95% confidence intervals) in blood pressure (SBP, DBP, MAP, PP) related to joint effects of daily walking distance volume and variability. Daily walking distance was categorised based on the terciles of the whole sample: low (daily step counts 4.5 km/day); medium (4.5 km/day to 7 km/day); high (7 km/day). Walking distance variability was categorised into: regular (SD of daily walking distance < 1.5 km/day) and irregular (1.5 $\geq$km/day). Models were adjusted for age, sex, body mass index, smoking status, drinking status, presence of hypertension, presence of type 2 diabetes, season, type of the day (weekday/weekend), and long term trend. SBP, systolic blood pressure; DBP, diastolic blood pressure; MAP, mean arterial pressure; PP, pulse pressure.


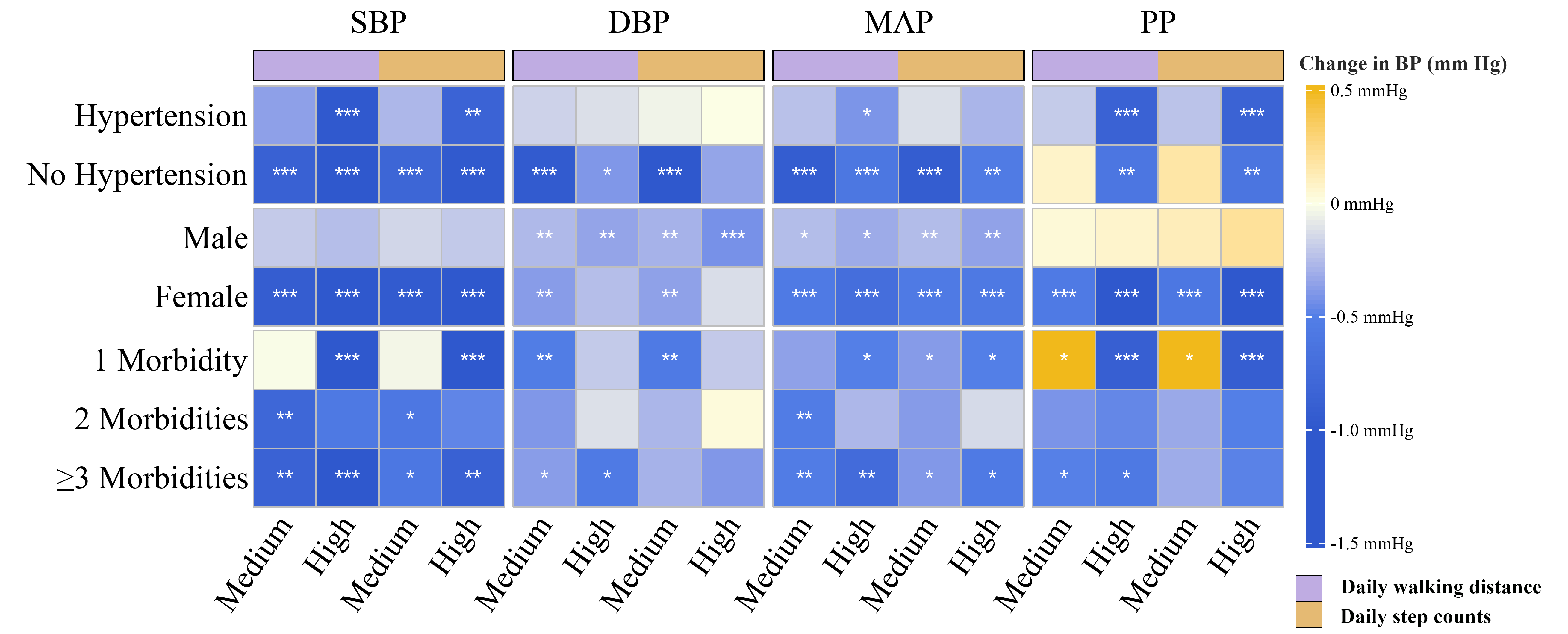


Figure S3. Associational patterns for daily walking distance and daily step counts with blood pressure (SBP, DBP, MAP and PP) across gender, presence of hypertension, and chronic disease status. Heatmap illustrated estimated change in BP for the effects of waking distance (indicated as purple bar) and step counts (indicated as yellow bar) across different sub-groups. Daily walking distance and daily step counts were categorized as low, medium, and high based on the tercile distributions of specific sub-population (Supplemental Table S1). * *P*<0.05, ** *P* <0.01, *** *P* < 0.001. SBP, systolic blood pressure; DBP, diastolic blood pressure; MAP, mean arterial pressure; PP, pulse pressure.
